# Oral Immunization with a Multivalent Epitope-Based Vaccine, Based on NAP, Urease, HSP60, and HpaA, Provides Therapeutic Effect on *H. pylori* Infection in Mongolian gerbils

**DOI:** 10.3389/fcimb.2017.00349

**Published:** 2017-08-04

**Authors:** Le Guo, Hua Yang, Feng Tang, Runting Yin, Hongpeng Liu, Xiaojuan Gong, Jun Wei, Ying Zhang, Guangxian Xu, Kunmei Liu

**Affiliations:** ^1^Ningxia Key Laboratory of Clinical and Pathogenic Microbiology, General Hospital of Ningxia Medical University Yinchuan, China; ^2^Department of Medical Laboratory, School of Clinical Medicine, Ningxia Medical University Yinchuan, China; ^3^Ningxia Key Laboratory of Cerebrocranial Diseases, Ningxia Medical University Yinchuan, China; ^4^Research Center for High Altitude Medicine, Qinghai University Xining, China; ^5^Medical School of Nantong University, Nantong University Nantong, China; ^6^Department of Molecular Microbiology and Immunology, Johns Hopkins University Baltimore, MD, United States

**Keywords:** *Helicobacter pylori*, multivalent epitope-based vaccine, therapeutic vaccine, urease, NAP, HpaA, HSP60

## Abstract

Epitope-based vaccine is a promising strategy for therapeutic vaccination against *Helicobacter pylori* (*H. pylori*) infection. A multivalent subunit vaccine containing various antigens from *H. pylori* is superior to a univalent subunit vaccine. However, whether a multivalent epitope-based vaccine is superior to a univalent epitope-based vaccine in therapeutic vaccination against *H. pylori*, remains unclear. In this study, a multivalent epitope-based vaccine named CWAE against *H. pylori* urease, neutrophil-activating protein (NAP), heat shock protein 60 (HSP60) and *H. pylori* adhesin A (HpaA) was constructed based on mucosal adjuvant cholera toxin B subunit (CTB), Th1-type adjuvant NAP, multiple copies of selected B and Th cell epitopes (UreA_27–53_, UreA_183–203_, HpaA_132–141_, and HSP60_189–203_), and also the epitope-rich regions of urease B subunit (UreB_158–251_ and UreB_321–385_) predicted by bioinformatics. Immunological properties of CWAE vaccine were characterized in BALB/c mice model. Its therapeutic effect was evaluated in *H. pylori*-infected Mongolian gerbil model by comparing with a univalent epitope-based vaccine CTB-UE against *H. pylori* urease that was constructed in our previous studies. Both CWAE and CTB-UE could induce similar levels of specific antibodies against *H. pylori* urease, and had similar inhibition effect of *H. pylori* urease activity. However, only CWAE could induce high levels of specific antibodies to NAP, HSP60, HpaA, and also the synthetic peptides epitopes (UreB_158–172_, UreB_181–195_, UreB_211–225_, UreB_349–363_, HpaA_132–141_, and HSP60_189–203_). In addition, oral therapeutic immunization with CWAE significantly reduced the number of *H. pylori* colonies in the stomach of Mongolian gerbils, compared with oral immunization using CTB-UE or *H. pylori* urease. The protection of CWAE was associated with higher levels of mixed CD4^+^ T cell (Th cell) response, IgG, and secretory IgA (sIgA) antibodies to *H. pylori*. *These results indic ate that a* multivalent epitope-based vaccine including Th and B cell epitopes from various *H. pylori* antigens could be a promising candidate against *H. pylori* infection.

## Introduction

*Helicobacter pylori* (*H. pylori*) is a helix-shaped bacterium that infects more than half of the world's population (Vakil et al., 2010). *H. pylori* infection is closely associated with gastritis, peptic ulcer disease, and stomach cancer (Parsonnet et al., 1991). Current antibiotic-based triple therapies have many disadvantages such as high cost, poor patient compliance, increasing antibiotic resistance, and reinfection (Graham and Fischbach, 2010). Therefore, antibiotic-based triple therapies are not practical for global control. Vaccination against *H. pylori* infection, especially therapeutic vaccination, could be an effective and economic strategy, either as an alternative or a complementary to antibiotic-based triple therapies.

Many antigens from *H. pylori*, such as urease, heat shock protein 60 (HSP60), *H. pylori* adhesin A (HpaA) and neutrophil-activating protein (NAP), have been proved to be the excellent candidates for their ability to induce protective immune responses against *H. pylori* infection (Satin et al., 2000; Yamaguchi et al., 2000; Lucas et al., 2001; Flach et al., 2011; Vermoote et al., 2013). NAP is not only a major virulence factor, but also a protective antigen. Besides, NAP has potential application as a general vaccine adjuvant for inducing Th1 cell-mediated immunity (D'Elios et al., 2007). HpaA is essential for the *adhesion* of *H. pylori* to human gastric tissue. It has been reported that a lysine rich peptide fragment from HpaA is involved in receptor recognition, which is crucial for the binding of *H. pylori* to gastric epithelium (Chaturvedi et al., 2001). *H. pylori* produces large amounts of urease (Ure) which is composed of two subunits, UreA and UreB. Urease can hydrolyze urea to ammonia and carbon dioxide, thereby neutralizing gastric acid and facilitating *H. pylori* colonization (Suerbaum and Josenhans, 1999). Many antigenic epitopes from *H. pylori* urease, such as Th cell epitope UreA_27–53_ (Rizos et al., 2003) and B cell epitopes UreA_183–203_ (Fujii et al., 2004) and UreB_321–339_ (Hirota et al., 2001), have been identified and could be useful for epitope-based vaccine development. The main heat shock proteins (HSP) possessed by *H. pylori* are the GroEL/S (58 KD also called HSPB/HSP60 and 13 KD also called HSPA, respectively) and the Dna K/J (also called HSP70) chaperones (Suerbaum et al., 1994). Heat shock protein 60 (HSP60) has been demonstrated to be expressed on the surface of *H. pylori*, and facilitate adhesion to host cells (Yamaguchi et al., 1997). The epitope peptide recognized by the H9 MAb against HSP60 was mapped to the sequence of amino acids 189–203 (HSP60_189–203_; Yamaguchi et al., 2000). A univalent vaccine composed of a single *H. pylori* antigen has limited protective efficiency against *H. pylori* infection. Therefore, a multivalent vaccine containing various antigens from *H. pylori* has been well accepted to be superior to a univalent vaccine (Corthesy et al., 2005; Wu et al., 2008). However, there are still some drawbacks in multivalent recombinant subunit vaccines containing several antigens. For example, each subunit antigen from the pathogen has a large molecular weight so that it is difficult to construct and express recombinant subunit vaccine containing more than two antigens. Therefore, it is an effective approach to construct multivalent epitope-based vaccines by using the selected epitope peptides or the predicted epitope-rich regions, instead of using the whole antigens.

Animal models have been widely emphused to study *H. pylori* infection, such as mice, rats, beagle dogs, cats, or nonhuman primates (Czinn and Blanchard, 2011). The most widely used animal model involves infection of mice with *H. pylori*. The mouse is small, inexpensive and convenient, and the elegant genetics permits molecular dissection of the host response to *H. pylori* infection. However, the function of the *H. pylori* Cag-type IV secretion system (T4SS) is commonly lost during colonization of mice (Philpott et al., 2002). This occurs less frequently in the *Mongolian gerbil* (*M. gerbil*), indicating the Mongolian gerbil model seems more suitable for *H. pylori* infection (Rieder et al., 2005). In addition, the M. gerbil is an efficient and cost-effective rodent model that recapitulates many features of *H. pylori*-induced gastritis and carcinogenesis in humans (Liu et al., 2016; Jang et al., 2017).

In this study, our aim is to develop a multivalent epitope-based vaccine, CWAE against *H. pylori*, involving urease A and B subunit (UreA and UreB), NAP, HSP60, and HpaA, which are involved in the adhesion and virulence of *H. pylori* to gastric mucosa. Immunological properties of CWAE vaccine were evaluated in BALB/c mouse model, and its therapeutic effect was analyzed in Mongolian gerbils, in which *H. pylori* infection is similar to that in humans.

## Materials and methods

### Multivalent epitope-based vaccine design

Based on a consensus approach which combines NN-align, SMM-align, and combinatorial library methods, potential immunodominant CD4^+^ T cell epitopes binding to human leukocyte antigen (HLA) class II molecules were screened from UreB by online T Cell Epitope Prediction Tools (IEDB Analysis Resource, http://tools.iedb.org/main/tcell/). The predicted CD4^+^ T cell epitopes with percentile rank ≤ 1 are considered high affinity and used as the candidate epitopes for vaccine design (Supplementary Data Sheet 1). At the same time, based on sequence characteristics of the UreB antigen using amino acid scales and Hidden Markov Model (HMM), potential linear B cell epitopes was screened by online B Cell Epitope Prediction Tools (IEDB Analysis Resource, http://tools.iedb.org/main/bcell/). An output threshold of 0.9 was used (sensitivity = 0.25, specificity = 0.91) for identification of linear B cell epitopes. This threshold was chosen to provide a high level of certainty for predicted B-cell epitopes from UreB. The predicted B cell epitopes are as shown in Supplementary Data Sheet 2. In addition, the known epitopes of UreB were also obtained from Immune Epitope Database (IEDB, http://www.iedb.org/home_v3.php), as shown in Supplementary Data Sheet 3. The final epitope-rich regions of UreB were determined by overall consideration on the predicted and known epitopes. Finally, the predicted epitope-rich regions (UreB_158–251_ and UreB_321–385_) and the other four B or Th cell epitopes [UreA_27–53_ (Rizos et al., 2003), UreA_183–203_ (Hifumi et al., 2006), HpaA_132–141_ (Chaturvedi et al., 2001), and HSP60_189–203_ (Yamaguchi et al., 2000)] were used to construct multivalent epitope-based vaccine. The reasonable combination of mucosal adjuvant CTB, Th1-type immune adjuvant NAP, the linkers (KK, GS, GGG, DPRVPSS), the tandem copies of Th or B cell epitopes (UreA_27–53_, UreA_183–203_, HpaA_132–141_, and HSP60_189–203_) and two predicted UreB epitope-rich regions (UreB_158–251_ and UreB_321–385_) were determined by modeling and prediction using RANKPEP, molecular operating environment (MOE), and DNAstar software.

### Construction of multivalent epitope-based vaccine

To construct the vector expressing the multivalent epitope-based vaccine, a DNA fragment named WAE encoding NAP, multiple copies of selected B and Th cell epitopes (UreA_27–53_, UreA_183–203_, HpaA_132–141_, and HSP60_189–203_) and the UreB epitope-rich regions (UreB_158–251_ and UreB_321–385_) were synthesized and cloned into vector pETC containing cholera toxin B subunit (CTB) gene, generating the plasmid pETCWAE. The plasmid pETCWAE was transformed into *E. coli* BL21 for expression of the fusion protein CWAE. The CWAE protein was purified by Ni^2+^-NTA affinity chromatography according to according to the manufacturer's instructions.

### Western blot

Purified CWAE and CTB-UE, various *H. pylori* antigens (UreA, UreB, HpaA, HSP60, and NAP; Linc-Bio, Shanghai, China) were applied to 15% SDS-PAGE and transferred onto polyvinylidene difluoride membrane (PVDF, Millipore). Rabbit anti-*H. pylori* polyclonal antibody (Rabbit anti-Hp PcAb, Abace biology, Beijing, China) was used as primary antibody for CWAE and CTB-UE. Mice anti-CWAE polyclonal antibody (prepared by our laboratory) was for various *H. pylori* antigens (UreA, UreB, HpaA, Hsp60, and NAP). After washing, the membrane was then incubated with HRP-Goat Anti-Rabbit IgG (Proteintech) or HRP-Goat Anti-Mouse IgG (Proteintech). The positive signals were monitored using HRP-DAB Chromogenic Substrate Kit (Tiangen Biotech) according to manufacturer's reagent instructions.

### Immunization and infection

All animal experiments were approved by the Animal Ethical and Experimental Committee of Ningxia Medical University. Immunological properties of CWAE vaccine were characterized in BALB/c mice model. SPF BALB/c mice (male, 5–6 weeks old; *n* = 6) were immunized with 100 μg of the purified CWAE, CTB-UE, or CTB (Absin Bioscience Inc., Shanghai) by abdominal multipoint subcutaneous injection with Freund's adjuvant 3 times at 1 week's interval. The antigens in PBS without adjuvant were for the last booster immunization. Anti-serum were separated on the fifth day after the last booster and were used for detection of antibody level and specificity.

**Figure 1:**
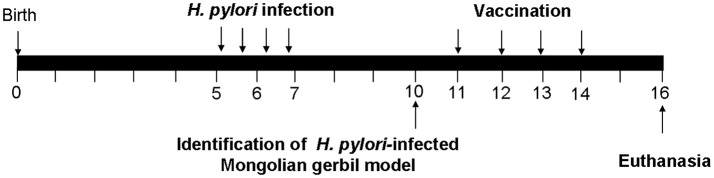
The experimental design of therapeutic vaccination. Briefly, Mongolian gerbils were infected with *H. pylori* SS1. We tested whether *H. pylori*-infected Mongolian gerbils are successful at the 10th week. *H. pylori*-infected Mongolian gerbils were administered orally with 100 μg of CWAE, CTB-UE, Urease in 500 μl aluminum hydroxide adjuvant on 4 occasions (weeks 11, 12, 13, and 14), and 500 μl PBS was also given orally on 4 occasions as control. Two weeks after the final immunization (At 16th weeks), Mongolian gerbils were sacrificed for various testing items.

Mongolian gerbils (male, 6 weeks old) were used for evaluating therapeutic effect of the CWAE vaccine. The experimental design of therapeutic vaccination was shown in Figure 1. Firstly, Mongolian gerbils were infected with *H. pylori* SS1 (10^9^ CFUs) using intubation, four times within the span of two weeks. For therapeutic vaccination, *H. pylori*-infected Mongolian gerbils were randomized into four groups (*n* = 7). Then *H. pylori*-infected Mongolian gerbils were vaccinated intra-gastrically with 100 μg of CWAE, CTB-UE or Urease in 500 μl aluminum hydroxide adjuvant for four times at 1-week interval. The infected Mongolian gerbils were also immunized with PBS, as control, using the same method. Two weeks after the final vaccination, Mongolian gerbils were sacrificed for evaluation of *H. pylori* infection.

### Assay for antigen-specific antibody and peptide-specific antibody

Serum antibody levels were measured by ELISA. ELISA plates were coated with 0.5 μg/well of large antigen (Urease, UreA, UreB, HSP60, HpaA, NAP, or *H. pylori* lysates) or with 1 μg/well of synthetic epitope peptide (UreA_183–203_, UreB_321–339_, UreB_158–172_, UreB_181–195_, UreB_211–225_, UreB_349–363_, HpaA_132–141_, and HSP60_189–203_). To measure the serum IgG, blood was collected immediately before sacrifice. The serum was isolated and serially diluted before assay. The titer was determined as the reciprocal of the last dilution, with an OD >2 times the negative specimens (normal mouse serum). To detect secretory IgA (sIgA), the supernatants from the homogenized stomach tissue, intestinal tissue or feces were collected and diluted 1:5 in PBS for analysis of sIgA.

### GM1-ELISA

The ability of the CTB component in CWAE to bind to its cellular receptor was assessed by GM1-ELISA as previously described (Areas et al., 2004). Briefly, ELISA plates were coated with 1 μg/well GM1 ganglioside or BSA for 24 h. After washing, ELISA plates were blocked by incubating with 5% (m/V) skim milk for 2 h. The CWAE, CTB-UE, CTB or UreB proteins (100 μg/ml) were then added to ELISA plates and incubated for 2 h. After that, a proper dilution of anti-CTB polyclonal antibody (Biomade Technology) was added to the plates and incubated for 1 h. After washing, HRP-conjugated goat anti-mouse IgG (Jackson ImmunoResearch, USA) was added to the plate and incubated for 1 h. Substrate tetramethylbenzidine (TMB, Tiangen Biotech) was then added and incubated for 10 min. The absorbance was measured at 450 nm.

### *H. pylori* urease neutralization assay

A protein A sepharose column (BioVision) was used to purify mouse IgG in the antiserum. *H. pyloti* urease were incubated with 50 μl serial dilutions of purified antiserum IgG (0–30 μg/ml) in ELISA plates for 12 h at 4°C. After that, The ELISA plates were incubated with 50 μl of 50 mM phosphate buffer (pH 6.8) containing 0.02% phenol red, 500 mM urea and 0.1 mM dithiothreitol (DTT) at 37°C. Color development was measured at 550 nm at 30 min intervals over a period of 3 h. Percentage inhibition was determined by the following equation: [(activity without antiserum − activity with antiserum)/(activity without antiserum) × 100].

### *H. pylori* quantification and urease activity determination

Two weeks after the final therapeutic vaccination, mice were killed for determination of the *H. pylori* colonization in the stomachs. Briefly, the stomach was dissected into two tissue fragments along the lesser curvature. One fragment was homogenized in 2 ml of PBS by using a tissue homogenizer. Serial 10-fold dilutions of the homogenate were plated on *H. pylori* selective plate (QingDao Hopebio Technology) supplemented with 7% goat blood, trimethoprim (5 μg/ml), polymixin B (5 μg/ml), and vancomycin (10 μg/ml) under microaerobic conditions. After 4–6 days culture, colonies were counted and the number of Colony-Forming Units (CFU) per stomach was calculated. The degree of *H. pylori* colonization in the mouse stomach was also measured by rapid urease test. Briefly, the antral portion of the stomach was immediately immersed in 500 μl of sodium phosphate buffer containing 500 mM urea, 0.02 % phenol red, and 0.1 mM DTT. The stomach sample was incubated at 37°C for 3 h. The supernatant of specimens was used for quantification at 550 nm (A550).

### Gastric histology

One strip of stomach tissue was cut out and fixed with formalin. Then, the stomach tissue was embedded in paraffin and stained with hematoxylin and eosin (HE). For evaluation of gastritis, the slides were “blinded” and the extent of gastritis was graded as follows: 0, none; 1, a few leukocytes scattered in the deep mucosa; 2, moderate numbers of leukocytes in the deep to mid mucosa and occasional neutrophils in the gastric glands (microabscesses); 3, dense infiltrates in the deep to mid mucosa, a few microabscesses; and 4, dense, diffuse infiltrates throughout the lamina propria and into the submucosa, frequent microabscesses. The stomach tissue sections were also assessed for the presence of *H. pylori* infection by immunohistochemical (IHC) staining using polyclonal anti-*H. pylori* antibody (Linc-Bio, Shanghai, China) and polymer-HRP based detection system (BioGenex).

### Specific T lymphocyte response and cytokine production

Splenic lymphocytes were isolated with lymphocyte separation medium (Dakewe Biotechnology Company) and cultured (2 × 10^5^ cells/well) with synthetic peptides of the Th epitopes (UreB_229–251_, UreA_27–53_, or UreB_373–385_), NAP, Urease or *H. pylori* lysates (5 μg/ml) in plates at 37°C for 72 h. Then, 10 μl of the CCK-8 solution (Dojindo Molecular Technologies Inc) was added into plates for 4 h. The results were expressed as stimulation indices (SI), defined as the index of lymphocyte proliferation according to formula: SI = the absorbance 450 value of stimulated cultures/the absorbance 450 value of negative control cultures. To determine cytokine production, culture supernatants from splenic lymphocytes stimulated by *H. pylori* lysates for 72 h were collected to assay for IL-4, IFN-γ, and IL-17 by using ELISA kits (Shanghai Jiang Lai Biotechnology Co. Ltd., China) according to the manufacturer's instructions.

### Statistical analyses

All data were analyzed with the GraphPad Prism 5 software and expressed as mean ± SD. Statistical significance was tested using Student's *t*-test. *p* < 0.05 was considered as statistically significant (^*^p < 0.05, ^**^p < 0.01, ^***^p < 0.001; ns, not significant).

## Results

### Design and construction of multivalent vaccine CWAE

**Figure 2:**
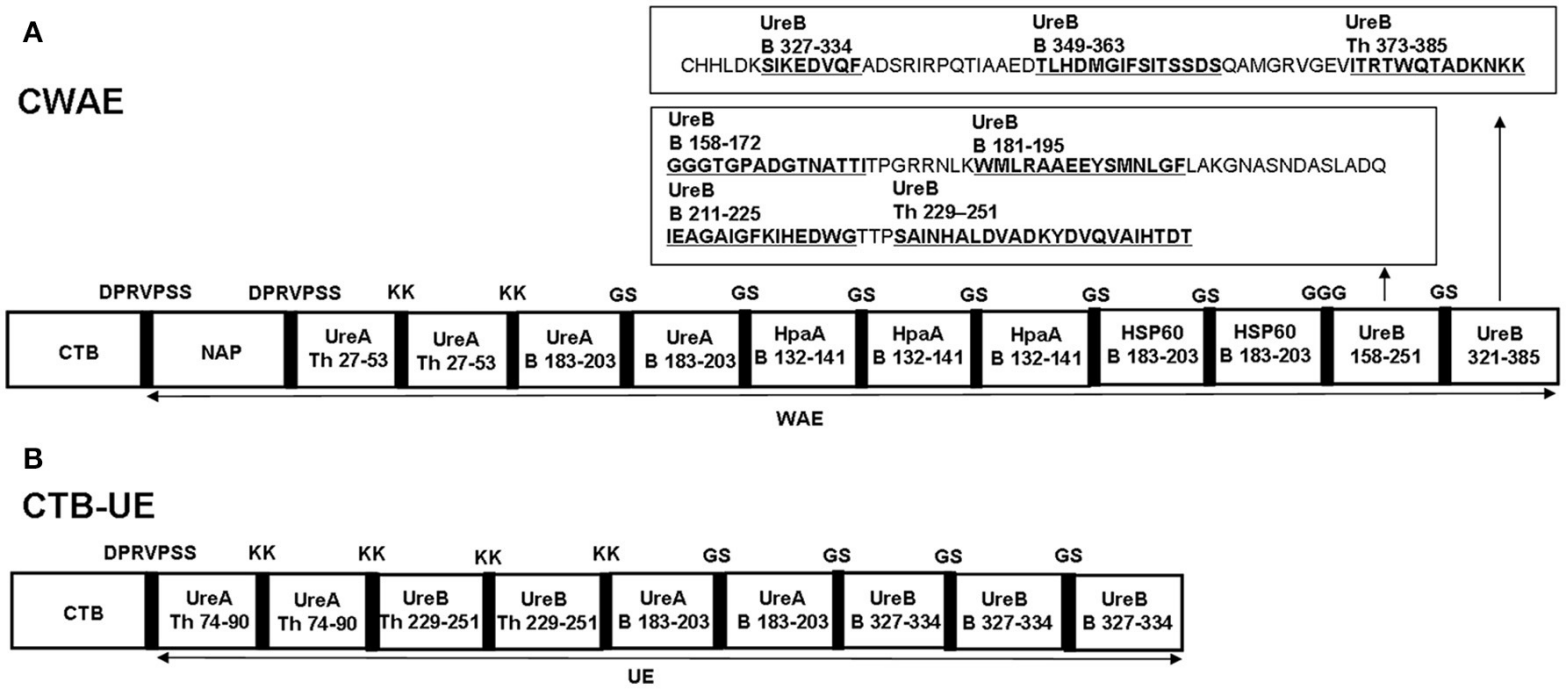
The structural diagrams of multivalent epitope-based vaccine CWAE and urease epitope-based vaccine CTB-UE. CWAE and CTB-UE share some same B or Th cell epitopes (UreB_229–25_, UreA_183–203_, and UreB_327–334_) which are marked in red fonts. **(A)** The CWAE vaccine is composed of two molecular adjuvants (CTB and NAP), tandem copies of the selected B and Th cell epitopes (UreA_27–53_, UreA_183–203_, HpaA_132–141_, and HSP60_189–203_), and epitope-rich regions UreB_158–251_ containing four known Th or B cell epitopes (UreB_158–172_, UreB_181–195_, UreB_211–225_, UreB_229–251_) and UreB_321–385_ containing three Th or B cell epitopes (UreB_327–334_, UreB_349–363_, UreB_373–385_). In order to avoid generating new epitopes at linkage sites, these linkers (DPRVPSS, KK, GGG, and GS) were designed to separate different epitopes or adjuvants. **(B)** The CTB-UE vaccine contains molecular adjuvants CTB and tandem copies of four different epitopes which are Th cell epitopes (UreA_74–90_ and UreB_229–251_) and B cell epitopes (UreA_183–203_ and UreB_327–334_) from *H. pylori* urease. In addition, the linkers (DPRVPSS, KK, and GS) were designed to retain the immunologic competence of each Th or B epitope and avoid the generation of new epitopes at linkage sites among epitopes.

An univalent epitope-based vaccine CTB-UE against *H. pylori* urease composed of molecular adjuvants CTB and tandem copies of urease B and Th cell epitopes was constructed in previous study (Guo et al., 2014), as shown in Figure 2B. In order to obtain a vaccine with better protective effect, a multivalent epitope-based vaccine named CWAE (GenBank access number. MF402943) against Urease (Ure), NAP, HSP60, and HpaA was constructed. The structure diagram of multivalent epitope vaccine CWAE is shown in Figure 2A. The epitope-rich regions UreB_158–251_ and UreB_321–385_ in CWAE were obtained by using online T Cell Epitope Prediction Tools and B Cell Epitope Prediction Tools. The epitope-rich region UreB_158–251_ was found to contain four known Th or B cell epitopes [UreB_158–172_ and UreB_181–195_ (Qiu et al., 2010), UreB_211–225_ (Li et al., 2008), UreB_229–251_ (Shi et al., 2007)]. The epitope-rich region UreB_321–385_ was also found to contains three known Th or B cell epitopes [UreB_327–334_ (Hirota et al., 2001), UreB_349–363_ (Qiu et al., 2010), and UreB_373–385_ (Yang et al., 2013)]. In addition, the other four B or Th cell epitopes UreA_27–53_ (Rizos et al., 2003), UreA_183–203_ (Hifumi et al., 2006), HpaA_132–141_ (Chaturvedi et al., 2001), and HSP60_189–203_ (Yamaguchi et al., 2000) were also used as the components of multivalent vaccine. The theoretically optimal combination of cholera toxin B subunit (CTB), NAP, linkers, the tandem copies of the selected epitopes, and epitope-rich regions of UreB was determined by modeling and prediction using RANKPEP, MOE, and DNAstar software. CTB and NAP were selected as intra-molecular adjuvants, and linker (KK, GS, GGG, DPRVPSS) was used as a spacer between epitope tandem. In order to construct the recombinant expression vector pETCWAE containing the fusion gene CWAE, a synthetic WAE gene was synthesized and subcloned into pETC, as shown in Supplementary Figure 1a. The recombinant vectors pETC and pETCWAE was identified by enzyme restriction (Supplementary Figures 1b,c) and confirmed by DNA sequencing (data not shown).

### Expression, purification, and antigenicity of CWAE

**Figure 3:**
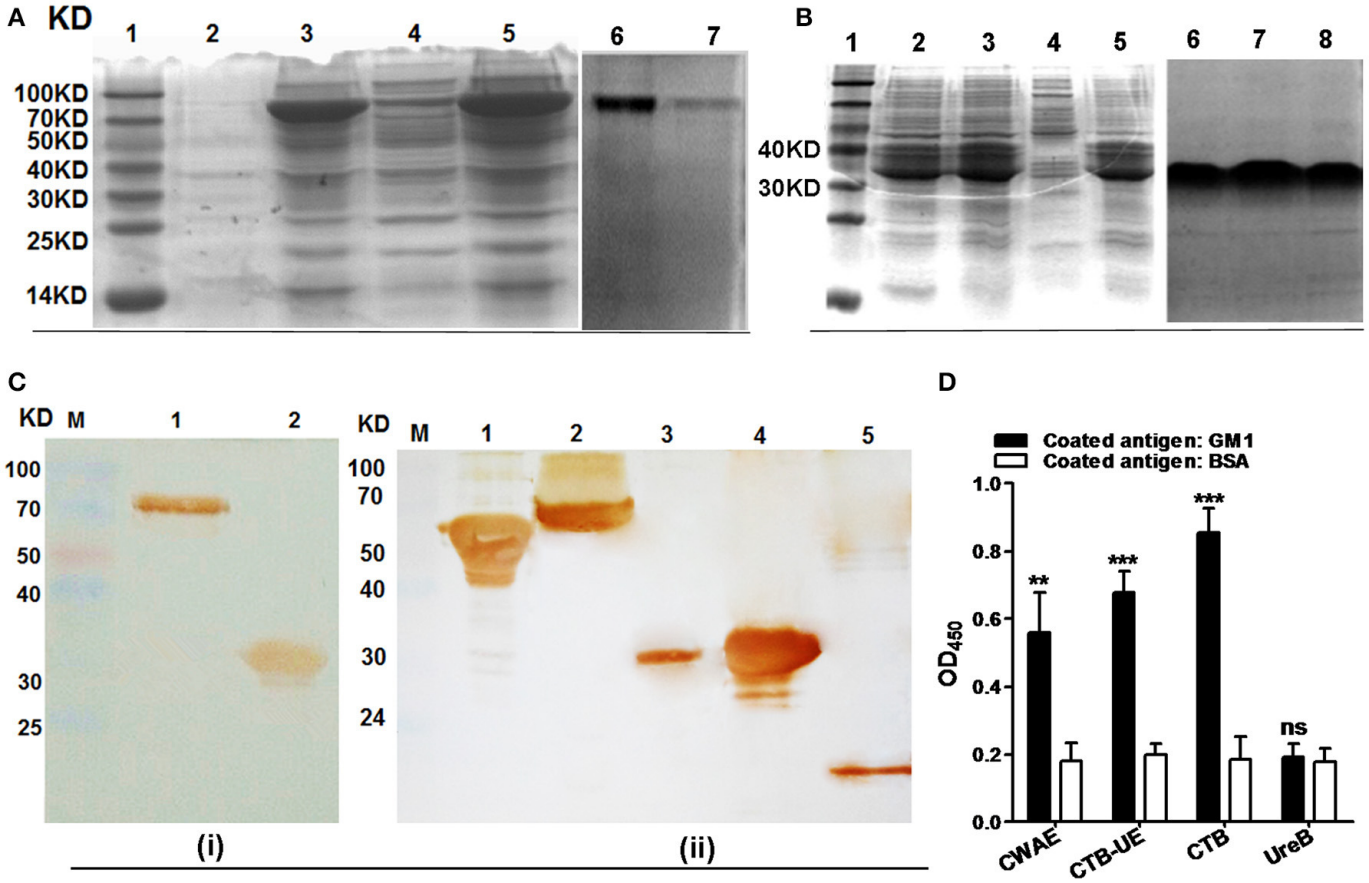
CWAE expression, purification, and antigen characteristics. **(A)** Protein expression and purification of CWAE. (Lane 1) protein marker, (lanes 3 and 5) the inclusion bodies of *E. coli* BL21(DE3)/pETCWAE, (lanes 2 and 4) the soluble proteins of *E. coli* BL21(DE3)/pETCWAE, (lane 6 and 7) the purified CWAE proteins. **(B)** Protein expression and purification of CTB-UE. (Lane 1) protein marker, (lanes 2, 3, and 5) the inclusion bodies of *E. coli* BL21(DE3)/pETCUE, (lane 4) the soluble proteins of *E. coli* BL21(DE3)/pETCUE, (lane 6, 7, and 8) the purified CTB-UE proteins. **(C)** Immunogenicity and immunoreactivity of CWAE analyzed by Western blot. **(i)** CWAE and CTB-UE reaction with Rabbit anti-*H. pylori* polyclonal antibody (Rabbit anti-Hp PcAb). (Lane M) protein marker, (lane 1) CWAE proteins, (lane 2) CTB-UE proteins. **(ii)** The *H. pylori* antigens (UreA, UreB, HpaA, Hsp60 and NAP) reaction with antiserum induced by CWAE vaccine. (Lane M) protein marker; (lane 1, 60 KD) Hsp60; (lane 2, 64 KD) UreB; (lane 3, 30 KD) UreA; (lane 4, 31 KD) HpaA; (lane 5, 14 KD) NAP. **(D)** The adjuvanticity of CTB component in CWAE and CTB-UE vaccine analyzed by GM1-ELSIA. In order to confirm the CTB component in CWAE or CTB-UE with the ability to bind GM1 gangliosides, GM1-ELISA was performed. ELISA plates were coated with 1 μg/well of GM1 ganglioside or BSA. The recombinant proteins CWAE, CTB-UE, CTB, and UreB with a concentration of 100 μg/ml were used to evaluate their capability of binding GM1. Data are mean ± *SD*. *p* < 0.05 was considered as statistically significant. ^**^p < 0.01; ^***^p < 0.001; ns, not significant.

**Figure 4:**
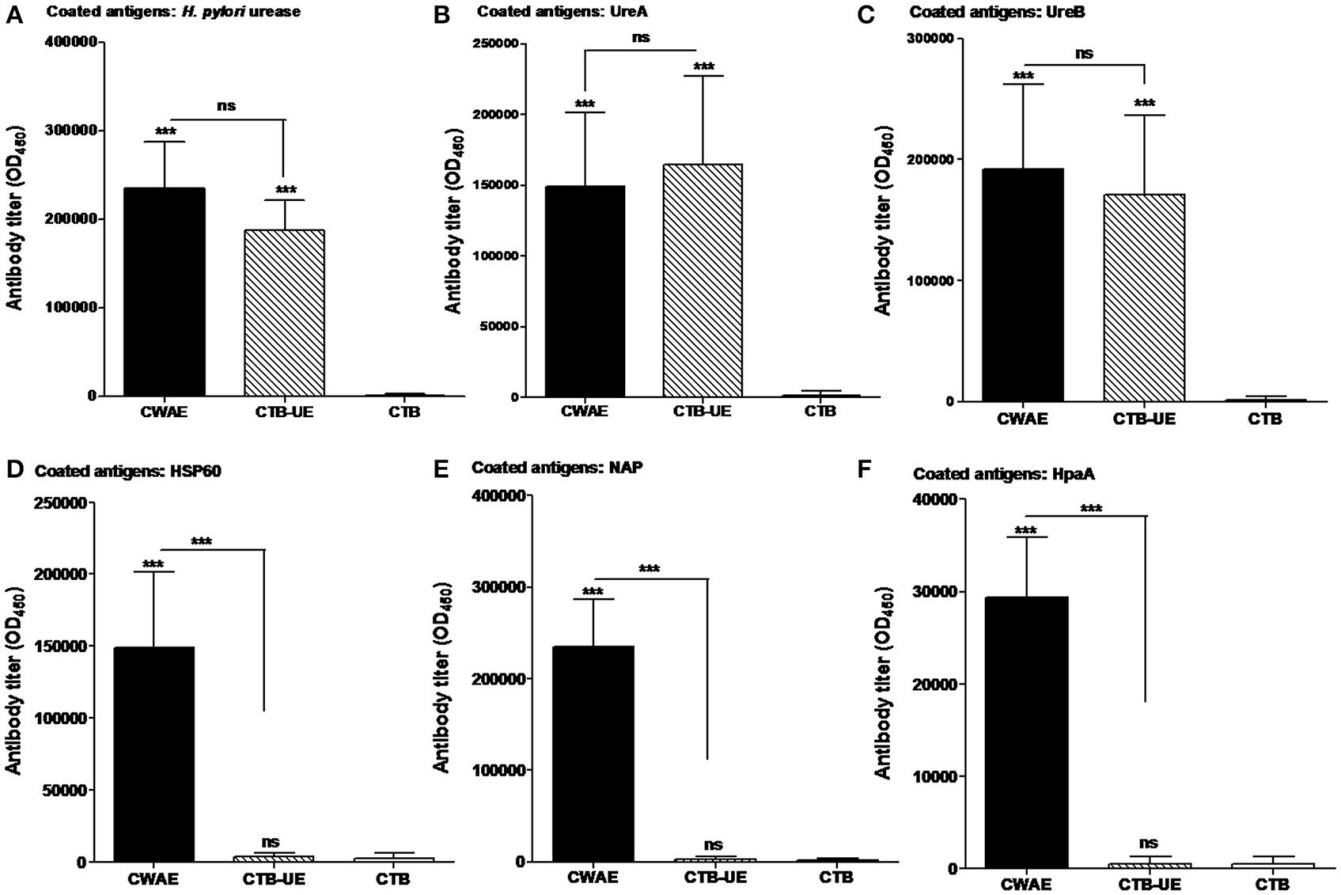
Assessment of antibodies specific for large antigens (*H. pylori* urease, UreA, UreB, Hsp60, NAP, and HpaA). The SPF BALB/c mice were immunized with CWAE, CTB-UE, or CTB by subcutaneous multi-point injection. **(A)** Detection of antibodies specific for *H. pylori* urease. ELISA plates were coated with 0.5 μg/well of native *H. pylori* urease. *p* <0.05 was considered as statistically significant. ^***^p < 0.001, ns, not significant. **(B)** Detection of antibodies specific for UreA. ELISA plates were coated with 0.5 μg/well of UreA. *p* < 0.05 was considered as statistically significant. ^***^p < 0.001, ns, not significant. **(C)** Detection of antibodies specific for UreB. ELISA plates were coated with 0.5 μg/well of UreB. *p* < 0.05 was considered as statistically significant. ^***^p < 0.001, ns, not significant. **(D)** Detection of antibodies specific for Hsp60. ELISA plates were coated with 0.5 μg/well of Hsp60. *p* < 0.05 was considered as statistically significant. ^***^p < 0.001, ns, not significant. **(E)** Detection of antibodies specific for NAP. ELISA plates were coated with 0.5 μg/well of NAP. *p* < 0.05 was considered as statistically significant. ^***^p < 0.001, ns not significant. **(F)** Detection of antibodies specific for HpaA. ELISA plates were coated with 0.5 μg/well of HpaA. *p* < 0.05 was considered as statistically significant. ^***^p < 0.001, ns, not significant.

The CWAE protein (about 70 KD) was largely expressed in inclusion body of *E. coli* BL21(DE3)/pETCWAE. Based on the SDS-PAGE, the content of CWAE in the inclusion bodies was about 60% of the total protein (Figure 3A; Lanes 3 and 5). After purification by Ni^2+^-NTA affinity chromatography, the purity of the CWAE protein was 95.8% as analyzed by SDS-PAGE (Figure 3A; Lanes 6 and 7) and computer scan. Besides, the CTB-UE protein was also mainly expressed in inclusion body (Figure 4B; Lane 2, 3, and 5) and had a relative high level of expression. The purity of the CWAE protein was 96.2% as analyzed by SDS-PAGE (Figure 3B; Lane 6, 7, and 8) and computer scan. The antigenic characteristics of CWAE and CTB-UE was identified by Western blot and GM1-ELSIA. Both CWAE and CTB-UE protein could be recognized by rabbit anti-*H. pylori* polyclonal antibody (Figure 3Ci). Similarly, the polyclonal antibody induced by the CWAE protein could react with *H. pylori* UreA, UreB, HpaA, Hsp60, and NAP (Figure 3Cii). The adjuvanticity of CTB component was analyzed by GM1-ELSIA. When GM1 was used as the coating protein, both CWAE and CTB-UE were able to bind GM1 (Figure 3D), but the ability of CWAE and CTB-UE to combine GM1 decreased compared with the positive control CTB. In addition, UreB could not bind GM1.

### Antigen-specific antibodies induced by CWAE vaccine

**Figure 5:**
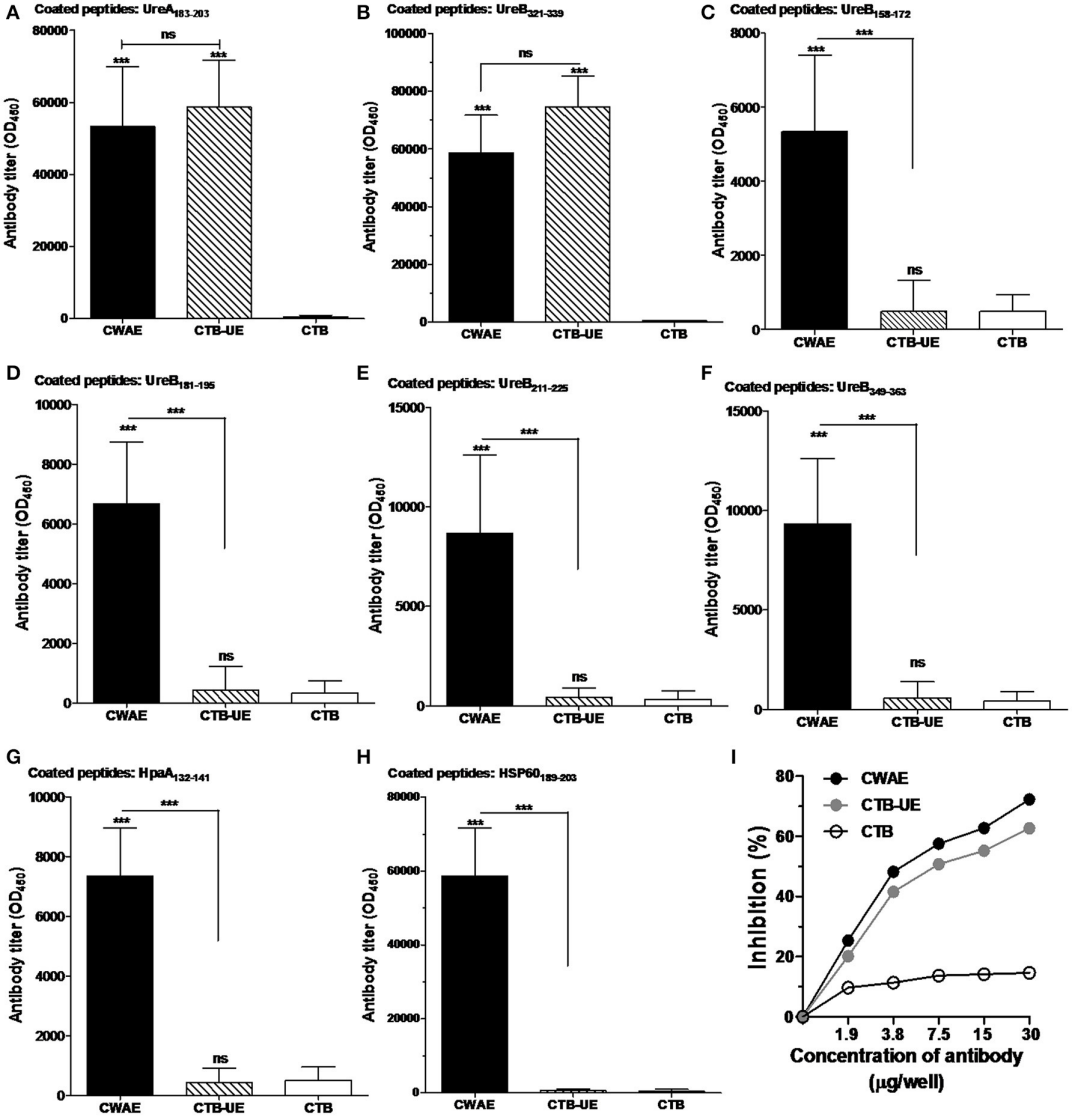
Detection of epitope-specific antibodies. The epitope peptides UreA_183–203_, UreB_321–339_, UreB_158–172_, UreB_181–195_, UreB_211–225_, UreB_349–363_, HpaA_132–141_, and HSP60_189–203_ were synthetized. Data are mean ± S.D. *p* < 0.05 was considered as statistically significant. ^***^p < 0.001, ns, not significant. **(A)** Measurement of antibodies specific for the UreA_183–203_ peptide. ELISA plates were coated with 1 μg/well of synthetic UreA_183–203_ peptides. **(B)** Measurement of antibodies specific for the UreB_321–339_ peptide. *ELISA plates were coated with* 1 μg/well of synthetic UreB_321–339_ peptides. **(C)** Measurement of antibodies specific for the UreB_158–172_ peptide. ELISA plates were coated with 1 μg/well of synthetic UreB_158–172_ peptides. **(D)** Measurement of antibodies specific for the UreB_181–195_ peptide. ELISA plates were coated with 1 μg/well of synthetic UreB_181–195_ peptides. **(E)** Measurement of antibodies specific for the UreB_211–225_ peptide. ELISA plates were coated with 1 μg/well of synthetic UreB_211–225_ peptides. **(F)** Measurement of antibodies specific for the UreB_349–363_ peptide. ELISA plates were coated with 1 μg/well of synthetic UreB_349–363_ peptides. **(G)** Measurement of antibodies specific for the HpaA_132–141_ peptide. ELISA plates were coated with 1 μg/well of synthetic HpaA_132–141_ peptides. **(H)** Measurement of antibodies specific for the HSP60_189–203_ peptide. ELISA plates were coated with 1 μg/well of synthetic HSP60_189–203_ peptides. **(I)** Inhibition of *H. pylori* urease activity by specific antibodies. Natural *H. pylori* urease was preincubated with a serial dilution of IgG from mice immunized with CWAE, CTB-UE, or rCTB. The optical density of the mixture was determined at 550 nm by the indicator of phenol red. The data are expressed as percentage inhibition.

Mice were killed for determination of antibody level and specificity. Antibodies specific for large antigens (*H. pylori* urease, UreA, UreB, HSP60, HpaA, and NAP) were measured by ELISA. CWAE and CTB-UE could induce similar levels of antibodies specific to *H. pylori* urease, UreA, or UreB (Figures 4A–C). In addition, the CWAE vaccine could induce antibodies specific for HSP60 (Figure 5D), NAP (Figure 4E), and HpaA (Figure 4F), but CTB-UE could not induce these specific antibodies, which confirmed that CWAE vaccine could mount antibody responses against various virulence factors and adhesion factors of *H. pylori*.

### Epitope-specific antibodies and anti-urease neutralizing antibodies

To examine the epitope-specific antibodies in mice induced by the CWAE vaccine, the B cell epitope peptides (UreA_183–203_, UreB_321–339_, UreB_158–172_, UreB_181–195_, UreB_211–225_, UreB_349–363_, HpaA_132–141_, and HSP60_189–203_) in the CWAE vaccine were synthesized and tested. The results indicated that both CWAE and CTB-UE vaccine were capable of generating similar levels of antibodies directed specifically against the epitope peptides UreA_183–203_ (Figure 5A) and UreB_321–339_ (Figure 5B). In addition, only CWAE vaccine could induce high levels of antibodies against UreB_158–172_ (Figure 5C), UreB_181–195_ (Figure 5D), UreB_211–225_ (Figure 5E), UreB_349–363_ (Figure 5F), HpaA_132–141_ (Figure 5G), and HSP60_189–203_ (Figure 5H). These results indicate that the B cell epitopes in CWAE vaccine retained their immunologic functions. To further confirm the effects of antibodies induced by CWAE and CTB-UE on *H. pylori* urease activity, a urease neutralization assay was performed. Natural *H. pylori* urease was incubated with a serial dilution of IgG induced by CWAE, CTB-UE, or rCTB. IgG antibodies induced by CWAE or CTB-UE could inhibit *H. pylori* urease activity dose-dependently, and the inhibitory effect of specific IgG from mice immunized with CWAE was stronger (Figure 5I). However, the IgG induced by CTB had no obvious inhibition. This result indicates that CWAE vaccine could induce higher levels of neutralizing antibodies against *H. pylori* urease than the whole urease antigen, which may be due to the B cell epitope UreA_183–203_, UreB_321–339_, UreB_158–172_, UreB_181–195_, UreB_349–363_, and UreB_211–225_ in CWAE vaccine.

### Analysis of bacterial colonization in the stomach of mice

**Figure 6:**
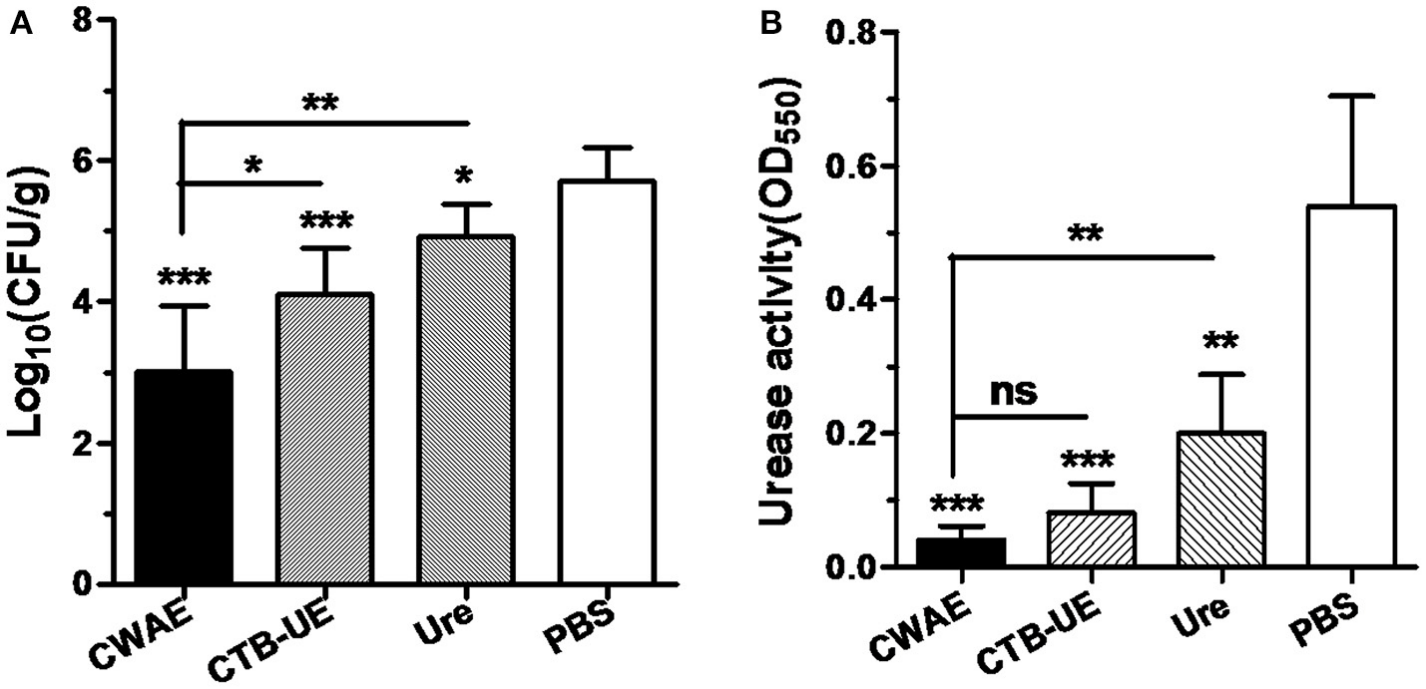
Evaluation of therapeutic effect by quantitative culture and rapid urease test. The *H. pylori*-infected Mongolian gerbils were orally immunized with CWAE, CTB-UE, Urease, or PBS. Data are mean ± S.D. *p* <0.05 was considered as statistically significant. ^*^p <0.05, ^**^p <0.01, ^***^p <0.001, ns, not significant. **(A)** Quantitative culture of *H. pylori* in the stomach after oral therapeutic immunization. The number of bacteria (CFU) per stomach was determined for individual mice in each group by quantitative culture. **(B)** The *H. pylori* urease activity in the stomach after oral therapeutic immunization. The *H. pylori* urease activity was measured by rapid urease test.

Since the CWAE vaccine showed good immunogenicity and immunological specificity, we next investigated whether oral immunization with CWAE vaccine could reduce the *H. pylori* load in the stomachs of Mongolian gerbils infected with *H. pylori*, and show better therapeutic effect than CTB-UE and Urease. Mongolian gerbils (already infected with *H. pylori*) were orally immunized with CWAE, urease or PBS. The *H. pylori* colonization in the stomach was analyzed by quantitative culture. The result showed that oral therapeutic immunization with CWAE, CTB-UE, or urease dramatically decreased the *H. pylori* loads in the stomachs of *H. pylori-*infected Mongolian gerbils compared with those using oral therapeutic immunization with PBS (Figure 6A). More importantly, the CWAE vaccine had a better reduction of bacterial burden than CTB-UE and Urease.

*H. pylori* can release large amounts of urease into the stomach. Urease decomposes urea into ammonia, which neutralizes gastric acid. Therefore, *H. pylori* urease is critical for *H. pylori* survival in the stomach. The results of rapid urease test showed that oral therapeutic immunization with CWAE, CTB-UE or urease could dramatically reduce the urease activity in the stomach, and the CWAE vaccine had a better treatment effect in decreasing urease activity in the stomach than Urease (Figure 6B). Unfortunately, there were not significant differences in urease activity between CWAE and CTB-UE vaccine, though the urease activity in the stomachs of Mongolian gerbils immunized with CWAE was lower than that of Mongolian gerbils immunized with CTB-UE.

### Histological analysis

**Figure 7:**
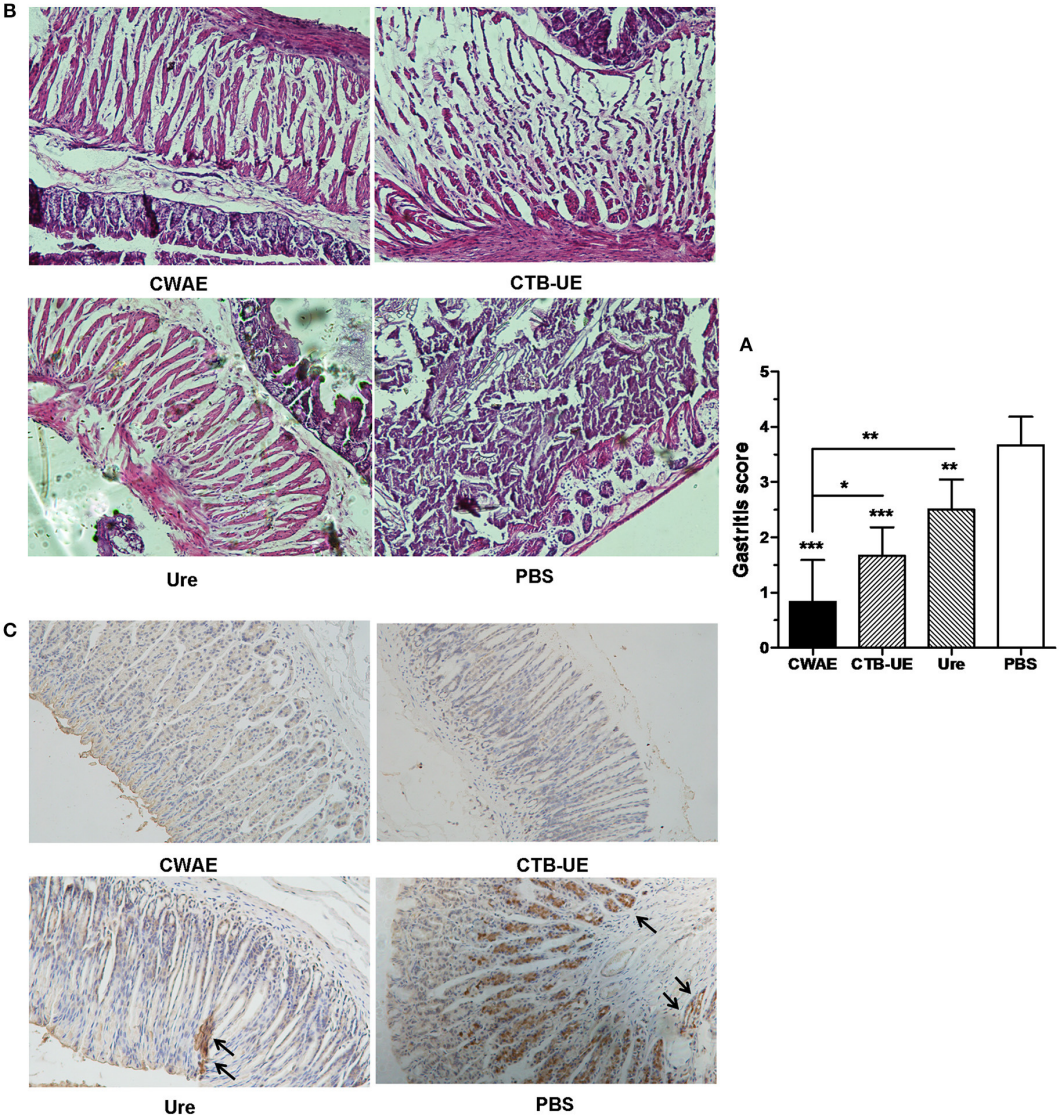
Histological analysis of stomach tissue. The *H. pylori*-infected Mongolian gerbils were orally immunized with CWAE, CTB-UE, Urease, or PBS. Data are mean ± S.D. *p* <0.05 was considered as statistically significant. ^*^p < 0.05, ^**^p < 0.01, ^***^p < 0.001, ns, not significant. **(A)** Assessment on therapeutic efficacy based on gastritis scores. The Inflammation score from Mongolian gerbils after immunization with CWAE is least. **(B)** Histopathological analysis after therapeutic vaccination. *H. pylori*-infected Mongolian gerbils after immunization with PBS showed severe inflammatory infiltrates (HE stain, 100×). However, *H. pylori*-infected Mongolian gerbils after immunization with CWAE, CTB-UE or Urease (Ure) showed mild inflammatory infiltrate. **(C)** Observation of *H. pylori* by IHC staining. The *H. pylori*-infected Mongolian gerbils after immunization with Urease (Ure) or PBS showed positively stained *H. pylori* within a glandular lumen. *H. pylori* colonizing in the stomach were denoted by black arrows.

Therapeutic effect of the CWAE vaccine was also analyzed by histopathological analysis of stomach tissue. High levels of leukocytes and neutrophils were found in the stomachs from *H. pylori*-infected Mongolian gerbils immunized with vehicle control PBS. In contrast, inflammation was weakened in the stomachs from *H. pylori*-infected Mongolian gerbils immunized with CWAE, CTB-UE, or Urease. Typical histological findings of gastric mucosa are shown in Figure 7A, and the results of histological scoring showed that the scoring grades of Mongolian gerbils immunized with CWAE were significantly lower than those of Mongolian gerbils immunized with Urease or CTB-UE (Figure 7B). Besides, Figure 7C show representative IHC results obtained with biopsies from mice immunized with CWAE, CTB-UE, Ure, and PBS, respectively. The IHC substantially confirmed the histopathologic observations.

### Antibody responses after therapeutic vaccination

**Figure 8:**
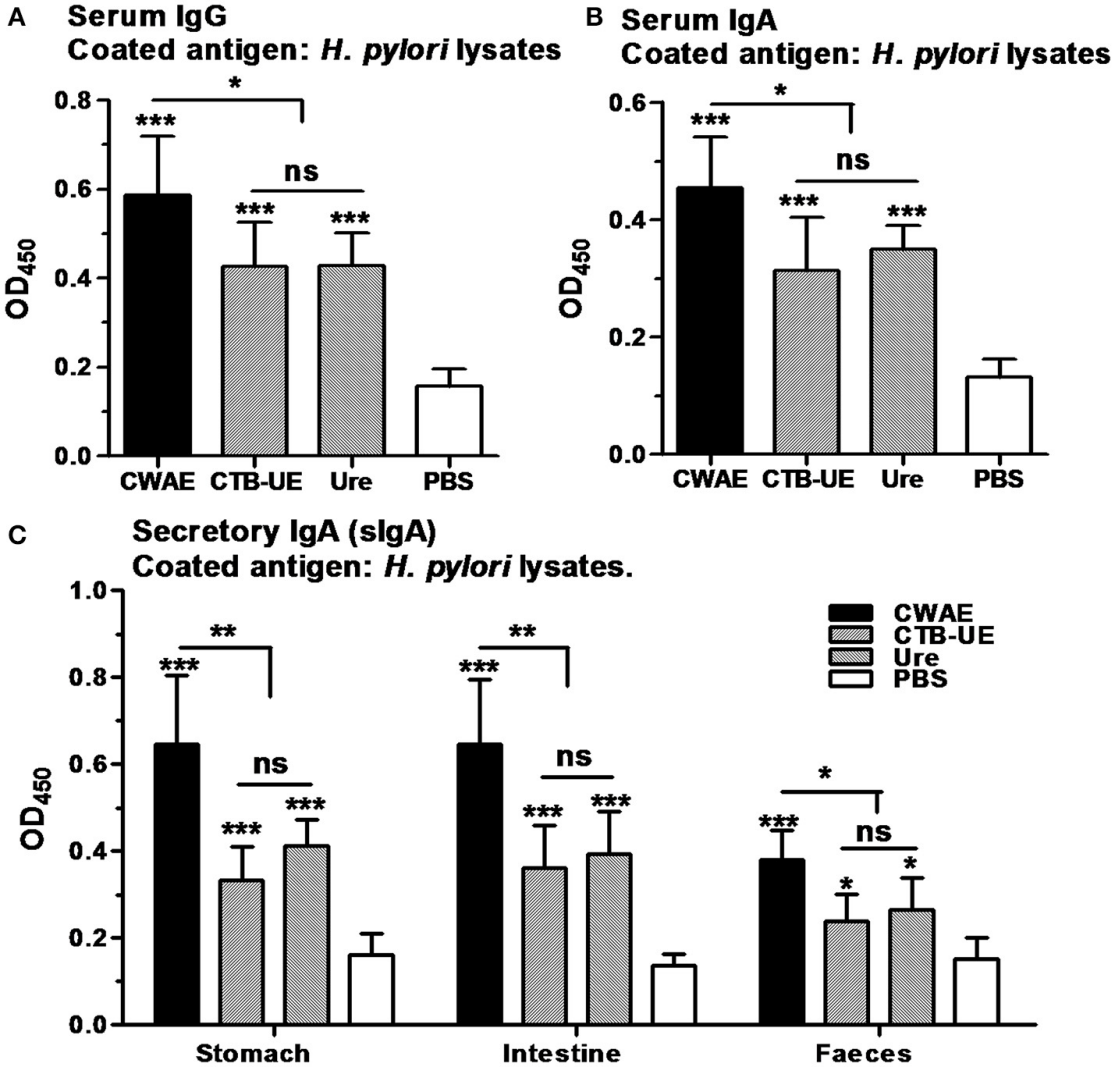
Antibody responses after oral therapeutic immunization. ELISA plates were coated with 0.5 μg/well of native *H. pylori* lysates. Data are mean ± S.D. *p* < 0.05 was considered as statistically significant. ^*^p <0.05, ^**^p < 0.01, ^***^p < 0.001, ns, not significant. **(A)** Detection of IgG against *H. pylori* lysates in serum. The different sera was diluted 1:800. **(B)** Detection of IgA against *H. pylori* lysates in serum. The different sera was diluted 1:800. **(C)** Detection sIgA against *H. pylori* lysates in the gastric mucus, intestinal mucus or feces. The supernatants from the homogenized stomach tissue, intestinal tissue or feces were diluted 1:5 in PBS for analysis of sIgA.

Antibody responses in serum, gastric and intestinal mucus, and feces were analyzed in Mongolian gerbils by ELISA after therapeutic vaccination. Oral immunization with CWAE, CTB-UE, or Urease significantly raised the levels of serum IgG and IgA against *H. pylori* lysates compared with oral immunization with PBS. In addition, the CWAE vaccine could induce higher levels of IgG and IgA specific for *H. pylori* lysates than Urease and CTB-UE (Figures 8A,B), which may be due to NAP component and the B cell epitopes from HSP60 and HpaA. Secretory IgA (sIgA) antibodies in gastric mucus, intestinal mucus and feces was also measured. A modest level of sIgA was found in extracts from the gastric mucus, intestinal mucus, or feces. Oral immunization with the CWAE, CTB-UE, or Urease remarkably increased the levels of sIgA antibodies against *H. pylori* lysates. Moreover, the CWAE vaccine could induce higher levels of sIgA specific for *H. pylori* lysates than Urease and CTB-UE (Figure 8C).

### Lymphocyte responses and cytokine production

**Figure 9:**
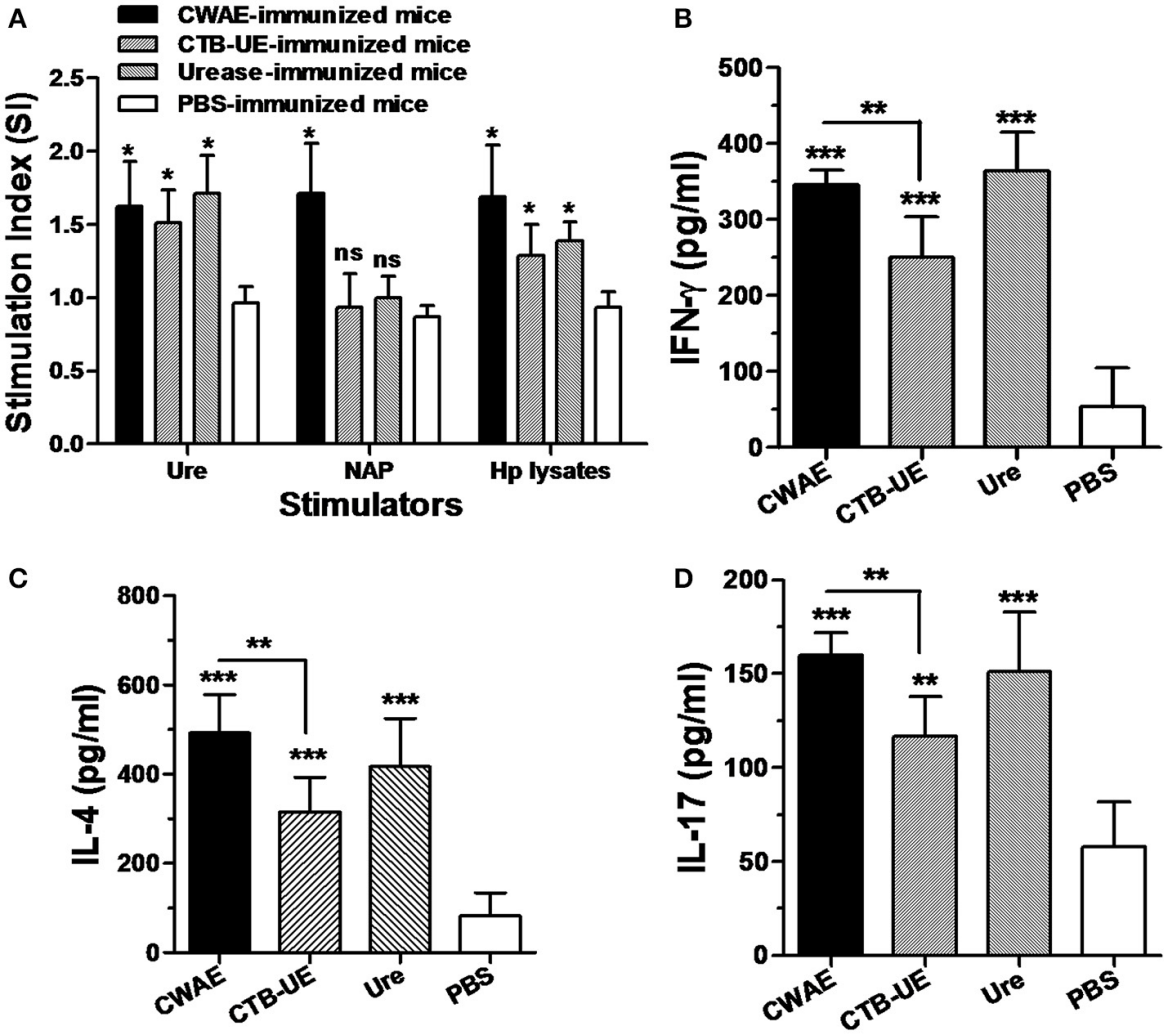
Lymphocyte responses and cytokine production after therapeutic vaccination. Data are mean ± *S.D*. *p* < 0.05 was considered as statistically significant. ^*^p < 0.05, ^**^p < 0.01, ^***^p < 0.001, ns, not significant. **(A)** Proliferation of splenic lymphocytes after stimulation with *H. pylori* antigens. Splenic lymphocytes were separated from *H. pylori*-infected Mongolian gerbils after therapeutic immunization with CWAE, CTB-UE, Urease, or PBS, and were incubated with NAP, Urease or *H. pylori* lysates (5 μg/ml). **(B–D)** Detection of IL-4, IFN-γ, and IL-17 cytokine production after therapeutic vaccination. The concentrations of cytokines in the supernatants of lymphocytes cultures were determined by ELISA. Splenic lymphocytes from *H. pylori*-infected Mongolian gerbils after therapeutic immunization with CWAE, CTB-UE, Urease, or PBS were stimulated with *H. pylori* lysates for 72 h, and cytokine production was detected by ELSA.

To determine the ability of CWAE to elicit lymphocyte specific responses for *H. pylori*, splenic lymphocytes after therapeutic immunization with CWAE, CTB-UE, Urease, or PBS were stimulated with NAP, Urease, or *H. pylori* lysates (Figure 9A). Splenic lymphocytes from Mongolian gerbils immunized with CWAE, displayed significantly high proliferation after stimulation with NAP, Urease or *H. pylori* lysates, compared with lymphocytes from Mongolian gerbils immunized with PBS. However, lymphocytes from Mongolian gerbils, immunized with Urease or CTB-UE after stimulation with *H. pylori* NAP, had no significant proliferation compared with cells from Mongolian gerbils immunized with PBS. These results showed that the multivalent vaccine CWAE could induce lymphocyte responses against various *H. pylori* antigens, and NAP component in CWAE kept the function as molecular adjuvant.

Cytokines IL-4, IFN-γ, and IL-17 in the supernatants of splenic lymphocyte cultures were measured by ELSIA after stimulation with *H. pylori* lysates. The splenic lymphocytes from Mongolian gerbils immunized with CWAE, CTB-UE, or *H. pylori* urease produced significantly high levels of IL-4, IFN-γ, and IL-17 cytokines. In addition, the CWAE vaccine could induce higher levels of IFN-γ (Figure 9B), IL-4 (Figure 9C), and IL-17 (Figure 9D) cytokines than CTB-UE, indicating that the CWAE vaccine induced comprehensive T cell responses of various types which were stronger than CTB-UE. However, there were no significant differences in the levels of IFN-γ, IL-4, and IL-17 cytokines between the CWAE- and Ure-immunized Mongolian gerbils.

## Discussion

That antibiotic treatment of *H. pylori* infection has many apparent shortcomings, such as increasing antibiotic resistance, reinfection and high cost calls for an effective and economic vaccine that can eradicate *H. pylori* infection. Various proteins from *H. pylori* have been demonstrated to be excellent candidate antigens in animal model, such as UreA (Lucas et al., 2001; Rizos et al., 2003; Guo et al., 2012), UreB (Guo et al., 2013; Vermoote et al., 2013; Zhang et al., 2014), HSP60 (Yamaguchi et al., 2000; Kamiya et al., 2002), HpaA (Nystrom and Svennerholm, 2007; Flach et al., 2011), and NAP (Satin et al., 2000; Rossi et al., 2004). More importantly, it has been reported that an oral recombinant subunit vaccine containing UreB and mucosal immune adjuvant LTA2B was found to be effective in *H. pylori*-naive children aged between 6 and 15 years, in a phase-3 clinical trial (Zeng et al., 2015). Although, the univalent vaccines against *H. pylori* infection have made significant progress, the multivalent vaccines may have more advantages and potential to induce more comprehensive protection against *H. pylori*, especially as therapeutic vaccines. However, it is difficult to obtain a safe and effective multivalent vaccine for *H. pylori* proteins with large molecular weight (MW) and biological toxicity. For example, the active *H. pylori* urease comprising two subunits, UreA (MW, 30 KD) and UreB (MW, 66 KD), has a molecular weight of about 96 KD and induces damage to epithelial cells (Rutherford, 2014). Epitope-based vaccines represent an attractive strategy for controlling *H. pylori* infection, which has many potential advantages including safety, preferable immunological specificity, and the opportunity to design a multivalent epitope-based vaccine with increased potency and more abroad spectrum (Sette and Fikes, 2003). In this study, we constructed a multivalent epitope-based vaccine CWAE against *H. pylori* utilizing urease, HSP60, HpaA, and NAP, which are critical for *H. pylori* colonization and virulence. We found that oral immunization with the multivalent epitope-based vaccine CWAE could induce high levels of epitope-specific antibodies against various *H. pylori* antigens, and significantly reduced *H. pylori* colonization in Mongolian gerbils, compared with CTB-UE or Urease. The immune protection of CWAE vaccine was correlated with high levels of antigen-specific Th cell responses and epitope-specific IgG, IgA, or sIgA antibodies.

Earlier studies have demonstrated that antibody responses against *H. pylori*, especially sIgA antibodies, contributed to the protective immunity in the mouse models (Ferrero et al., 1997; Nystrom and Svennerholm, 2007). However, some studies showed that antibodies are not essential for protective immunity against *H. pylori* (Ermak et al., 1998). We speculated that antibodies with high specificity against various key *H. pylori* antigens might exhibit a certain protection against *H. pylori*. It has been reported that a trivalent vaccine including CagA, VacA, and NAP could reduce *H. pylori* colonization and gastritis in *H. pylori*-infected Beagle dogs by intramuscular injection with aluminum hydroxide adjuvant, which may be due to antibody responses against CagA, VacA, and NAP (Rossi et al., 2004). It is likely that effective immunity against *H. pylori* is correlated to specific antibody responses against various *H. pylori* antigens participating in different aspects of the pathogenesis of *H. pylori* infection. In addition, polyclonal IgG antibodies induced by purified urease did not induce inhibitory effect on urease activity (Nagata et al., 1992). However, several monoclonal antibodies against urease could inhibit the enzymatic activity of *H. pylori* urease (Hirota et al., 2001; Fujii et al., 2004), and epitope-based vaccines which can induce those monoclonal antibodies possessed protective effect against *H. pylori*. Thus, epitope-specific antibodies may play a role in the protection. In our study, the multivalent vaccine CWAE could induce antibodies specific for urease, HSP60, HpaA, and NAP, which are critical for *H. pylori* colonization and pathogenicity. Moreover, these antibodies belong to epitope-specific antibodies against specific amino acid fragment of *H. pylori* antigens. For example, antibodies induced by CWAE could recognize the UreA_183–203_, UreB_321–339_, UreB_158–172_, UreB_181–195_, UreB_211–225_, UreB_349–363_ fragments which are closely correlated to the enzymatic activity of *H. pylori* urease (Hirota et al., 2001; Fujii et al., 2004; Qiu et al., 2010), the HpaA_132–141_ fragments involved in receptor recognition with gastric epithelium, and the HSP60_189–203_ fragments which have all been proven to induce a protective immune response against *H. pylori* (Yamaguchi et al., 2000).

A critical role of CD4^+^ T cells (Th cells) in protection against *H. pylori* has been widely accepted (Ermak et al., 1998). However, whether Th1, Th2, or Th17 responses play dominant role in the protective immunity against *H. pylori* remains controversial. There is some evidence supporting that Th2 cell responses dominate the protective effect against *H. pylori* infection (Mohammadi et al., 1997; Saldinger et al., 1998). However, recent studies demonstrate that Th1 or Th17 cells mediate protection (DeLyria et al., 2009; Li et al., 2015). Meanwhile, other studies suggested that the protective immunity against *H. pylori* was mediated by mixed Th cell responses (Liu et al., 2011; Chen et al., 2014; Yang et al., 2015). Generally speaking, the immune protective mechanism against *H. pylori* infection still needs to be elucidated for the complementarity and complex network of immunologic system. In our study, oral immunization with CWAE had a significant therapeutic effect on *H. pylori*-infected Mongolian gerbils. Furthermore, analysis of the cytokine production showed that IL-4, IFN-γ, and IL-17 were all significantly induced by CWAE, indicating that the CWAE vaccine stimulated mixed Th cell responses. The mixed Th cell response induced by CWAE may be related to Th1-type cellular immune adjuvant (NAP) and mucosal adjuvant (CTB) within the vaccine, aluminum hydroxide adjuvant, and peptide components of CWAE. Furthermore, the lymphocyte proliferation results showed that splenic lymphocytes from Mongolian gerbils immunized with CWAE proliferated significantly after stimulation with NAP, Urease, and *H. pylori* lysates, indicating the CWAE can also induce lymphocyte responses against various *H. pylori* antigen. Interestingly, CWAE and Urease vaccinations elicited comparable production of IFN-γ, IL-4, and IL-17, while CWAE had significantly higher protective efficacy compared to URE vaccine, suggested that many factors, not only Urease, may be involved in *H. pylori* infection *in vivo*, and multi-target intervention in the process of *H. pylori* infection is critical for eradicating *H. pylori* colonization. The CWAE vaccine composed of HLA restricted CD^4+^ T (Th) cell epitopes could induce Th cell responses against *H. pylori* in Mongolian gerbils, implying the predicted epitope-rich regions of UreB may also contain some Mongolian gerbil Th cell epitopes. Besides, the CWAE vaccine also contains a lot of B cell epitopes, which can induce epitope-specific antibodies against *H. pylori*. Therefore, the immune protection of CWAE vaccine was correlated with high levels of Th cell responses and epitope-specific IgG, IgA, or sIgA antibodies.

In conclusion, a multivalent epitope-based vaccine CWAE against *H. pylori* was designed and constructed in this study. Oral immunization with CWAE significantly reduced *H. pylori* colonization in *H. pylori*-infected Mongolian gerbils, which may be related to mixed Th cell responses and epitope-specific antibodies against various *H. pylori* antigens. Given the complex nature of *H. pylori* infection, the CWAE vaccine will face many challenges *on the road* to a human vaccine. We will further evaluate the therapeutic effect of CWAE in other animal models and investigate other oral immune delivery systems, such as polysaccharide microsphere and food grade lactic acid bacteria, to help CWAE to effectively override or bypass enzymatic degradation in the human gastrointestinal tract. Furthermore, clinical trials of the therapeutic multivalent epitope-based vaccine are expected in the future. This multivalent epitope-based vaccine may be a promising vaccine candidate that may help to control *H. pylori* infection.

## Author contributions

Conceived and designed the experiments: LG, KL, and GX. Performed the experiments: FT, RY, HY, XG, and HL. Analyzed the data: LG and FT. Contributed reagents/materials/analysis tools: JW. Wrote the manuscript: LG, YZ, and RY.

### Conflict of interest statement

The authors declare that the research was conducted in the absence of any commercial or financial relationships that could be construed as a potential conflict of interest.

## Supplementary material

Supplementary Data Sheet 1 The predicted CD4^+^ T cell epitopes of UreB. The predicted CD4^+^ T cell epitopes with percentile rank ≤ 1 are considered high affinity and used as the candidate epitopes for vaccine design.

Supplementary Data Sheet 2 The predicted B cell epitopes of UreB. An output threshold of 0.9 was used (sensitivity = 0.25, specificity = 0.91) for identification of linear B cell epitopes of UreB.

Supplementary Data Sheet 3 The known CD4^+^ T and B epitopes of UreB. The known epitopes of UreB were obtained from Immune Epitope Database.

Supplementary Figure 1 Construction and identification of recombinant vectors. **(a)** The schematic flow of expression vector construction. The vector pETCWAE expressing the CWAE protein was obtained by inserting the synthetical gene WAE into pETC vector containing CTB gene. **(b)** Identification of the pETC vector. 1: DNA marker; 2: the recombinant plasmid pETC digested by Nco I and Xho I; After digestion, a 342 bp DNA fragment was obtained, which was consistent with the theoretical size of CTB-L fusion gene. **(c)** Identification of the pETCWAE vector. 1: DNA marker; 2: the recombinant plasmid pETCWAE digested by Nco I and Xho I; After digestion, a 1797 bp DNA fragment was obtained, which was consistent with the theoretical size of CWAE gene.
