# Supplementary figures and images for: Oral Immunization with a Multivalent Epitope-Based Vaccine, Based on NAP, Urease, HSP60, and HpaA, Provides Therapeutic Effect on H. pylori Infection in Mongolian gerbils
Source: Front Cell Infect Microbiol. 2017 Aug 4;7:349. doi: 10.3389/fcimb.2017.00349 (PMC5543039; doi:10.3389/fcimb.2017.00349)

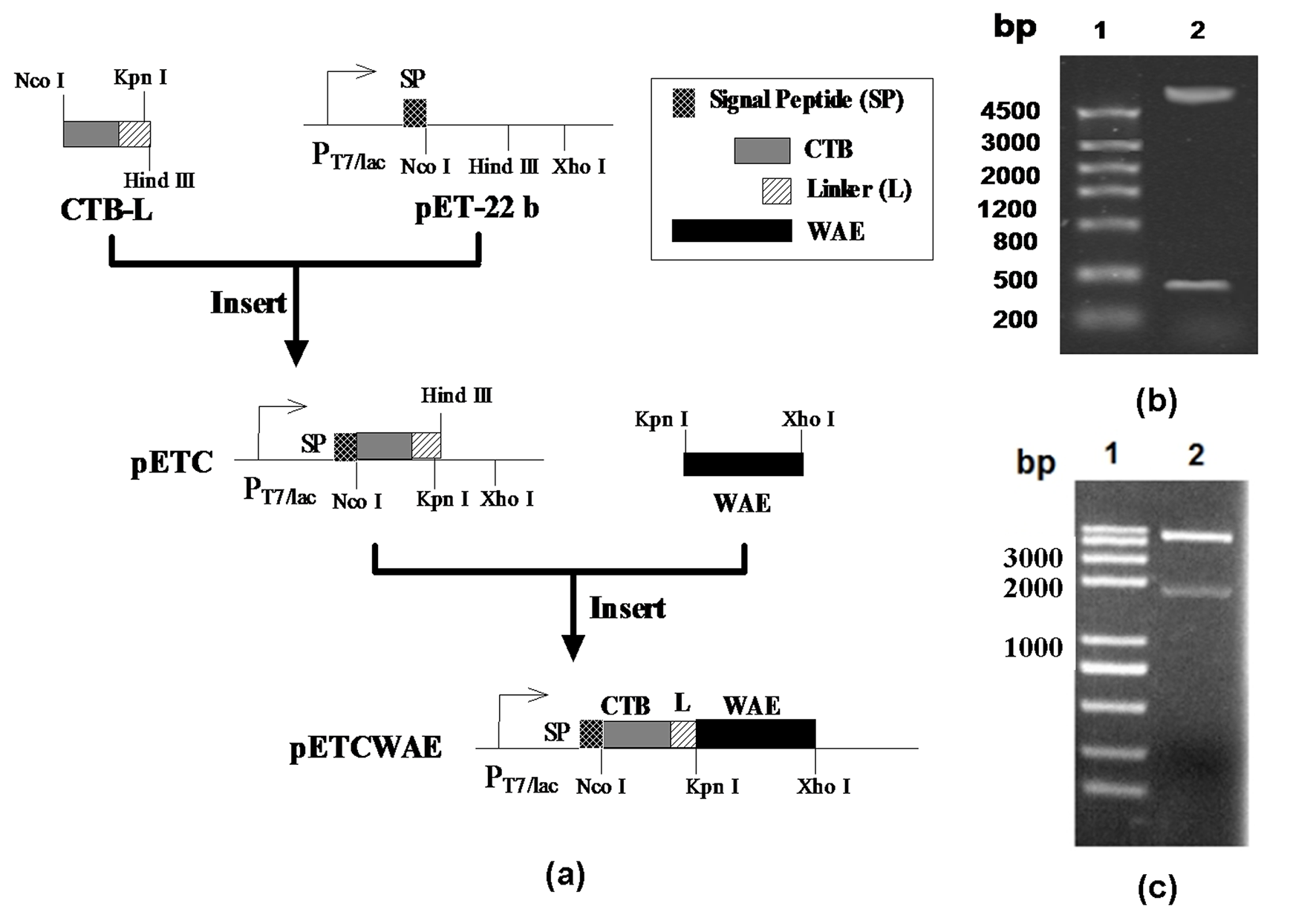

Supplement: Supplementary Figure 1 — Construction and identification of recombinant vectors. (a) The schematic flow of expression vector construction. The vector pETCWAE expressing the CWAE protein was obtained by inserting the synthetical gene WAE into pETC vector containing CTB gene. (b) Identification of the pETC vector. 1: DNA marker; 2: the recombinant plasmid pETC digested by Nco I and Xho I; After digestion, a 342 bp DNA fragment was obtained, which was consistent with the theoretical size of CTB-L fusion gene. (c) Identification of the pETCWAE vector. 1: DNA marker; 2: the recombinant plasmid pETCWAE digested by Nco I and Xho I; After digestion, a 1797 bp DNA fragment was obtained, which was consistent with the theoretical size of CWAE gene. [file Image1.TIF]
